# An innovative ensemble model based on deep learning for predicting COVID-19 infection

**DOI:** 10.1038/s41598-023-39408-8

**Published:** 2023-07-29

**Authors:** Xiaoying Su, Yanfeng Sun, Hongxi Liu, Qiuling Lang, Yichen Zhang, Jiquan Zhang, Chaoyong Wang, Yanan Chen

**Affiliations:** 1 School of Jilin Emergency Management, Changchun Institute of Technology, Changchun, 130021 China; 2 College of Computer Science and Technology, Jilin University, Changchun, 130012 China; 3 School of Environment, Northeast Normal University, Changchun, 130024 China

**Keywords:** Biological techniques, Computational biology and bioinformatics, Medical research

## Abstract

Nowadays, global public health crises are occurring more frequently, and accurate prediction of these diseases can reduce the burden on the healthcare system. Taking COVID-19 as an example, accurate prediction of infection can assist experts in effectively allocating medical resources and diagnosing diseases. Currently, scholars worldwide use single model approaches or epidemiology models more often to predict the outbreak trend of COVID-19, resulting in poor prediction accuracy. Although a few studies have employed ensemble models, there is still room for improvement in their performance. In addition, there are only a few models that use the laboratory results of patients to predict COVID-19 infection. To address these issues, research efforts should focus on improving disease prediction performance and expanding the use of medical disease prediction models. In this paper, we propose an innovative deep learning model Whale Optimization Convolutional Neural Networks (CNN), Long-Short Term Memory (LSTM) and Artificial Neural Network (ANN) called WOCLSA which incorporates three models ANN, CNN and LSTM. The WOCLSA model utilizes the Whale Optimization Algorithm to optimize the neuron number, dropout and batch size parameters in the integrated model of ANN, CNN and LSTM, thereby finding the global optimal solution parameters. WOCLSA employs 18 patient indicators as predictors, and compares its results with three other ensemble deep learning models. All models were validated with train-test split approaches. We evaluate and compare our proposed model and other models using accuracy, F1 score, recall, AUC and precision metrics. Through many studies and tests, our results show that our prediction models can identify patients with COVID-19 infection at the AUC of 91%, 91%, and 93% respectively. Other prediction results achieve a respectable accuracy of 92.82%, 92.79%, and 91.66% respectively, f1-score of 93.41%, 92.79%, and 92.33% respectively, precision of 93.41%, 92.79%, and 92.33% respectively, recall of 93.41%, 92.79%, and 92.33% respectively. All of these exceed 91%, surpassing those of comparable models. The execution time of WOCLSA is also an advantage. Therefore, the WOCLSA ensemble model can be used to assist in verifying laboratory research results and predict and to judge various diseases in public health events.

## Introduction

Public health events have become increasingly common since globalization, with COVID-19 being the most prominent example. However, due to the multiple mutations that COVID-19 undergoes during transmission, it is crucial to identify infections safely and efficiently. Previous research on predicting COVID-19 has primarily focused on monolithic neural network models^1,2^. ANN provides direction in the medical decision-making process. Researchers used an artificial neural network model to study the status of patients with COVID-19^3^. In addition, X-ray images are used as input data, and the CNN model is used to classify and evaluate the severity level of COVID-19 patients^4^. A monolithic model has low prediction accuracy. Although a small number of studies have used mixed models to predict the development trend of COVID-19^5–9^. Qu et al.^10^ proposed an innovative ensemble model that utilizes uses a new whale optimization algorithm (SCWOA) to predict COVID-19. This approach assigns different weights to each neural network model and optimizes the weight of the ensemble model. The results of the tests demonstrate that different deep learning models have varying prediction performance. Wang Gang compared the differences between CARIMA, es, GRNN and ARIMA–GRNN in predicting the number of daily cases. This result demonstrated that the ARIMA–GRNN mixed model^8^ is better. Mohan et al.^11^ used Supervised machine learning model (EAMA) for predicting long-term COVID-19 related parameters within India and globally. Kuvvetli et al.^7^ predicted the number of daily deaths and cases of COVID-19 with three different ANN models. To predict COVID-19 severity, logistic regression and ANN were applied to construct the COVID-19 severity prediction model^12^. Anum Shafiq et al.^13^ predict mortality rate of COVID-19 in Italy. Moreover, there are many methods for establishing prediction models in the medical field, yet they are only used in other aspects of medicine, which is different from the direction of this article using clinical data to study COVID-19^14–17^, such as epilepsy^18,19^, Cholera^20,21^, Ebola^22–25^. Machine learning provides a good effect and a reliable explanation for the study of diseases. Deep Learning as a Data-Dependent alternative to predict the development of the US COVID-19 epidemic^26^. Although these models have made significant contributions to epidemic prevention and control, existing research rarely uses laboratory indicators of patients to predict COVID-19 infection in biomedicine, and their prediction performance is poor. In this respect, in order to provide an accurate prediction model for medical institutions, we put forward an innovative deep learning model.

Optimizing and adjusting parameters using correlations between them is a critical step towards achieving optimal solutions for deep learning models^27–30^. Usually, the parameters are determined through a trial and error approach which is often fraught with errors and particularities, making it difficult to predict the approximate optimal solution in most cases, thus affecting the prediction performance of model. The Convolutional Neural Networks, Long-Short Term Memory (CNNLSTM) model^31^, which combines both spatial and temporal depth, holds significant promise. Brodzicki et al.^29^ has investigated the function of the whale optimization algorithm with a simple mechanism, few parameters, and strong optimization ability, and simulated the three-dimensional characteristics of the original Whale Optimization Algorithm (WOA). The results demonstrate that WOA is an excellent algorithm, that can optimize parameters more effectively than other well-known heuristic optimization algorithms.

This paper presents a new system for detecting COVID-19 by enhancing the original deep learning model. Based on the research of Alakus and Turkoglu^31^, we employ the Whale Optimization Algorithm to reconstruct the architecture of the CNNLSTM model and optimize its parameters. Therefore, a new ensemble model is proposed to predict COVID-19 infection. In this paper, key contributions can be summarized as the following:

- Optimization of the internal parameters of the CNNLSTM using WOA, and reconstructing its architecture. In addition, we use Grey Wolf Optimizer (GWO) to optimize the internal parameters of CNNLSTM, and take it as one of the comparative experiments of different deep learning models (GWOCLSA).
- The ensemble model (WOCLSA) is proposed to improve the prediction performance.
- The innovative ensemble model can be used to predict various public health diseases and provide help for medical disease diagnosis.

The structure of this study is as follows. Section "Methods" describes three neural network approaches, generalization of WOA and generalization of GWO. Section "Innovating the framework of forecasting model" describes three hybrid models as well as the WOCLSA. Section "Data description and evaluation criteria" summarizes the data sources and specific performance evaluation standards. Section "Experimental results and analysis" talks about the prediction results of the proposed model and compares the results with those of other models. Section "Discussion" is the discussion of this paper. At last, Section "Conclusion" is the conclusion of this paper.

## Methods

### CNN

CNN is a deep neural network model with convolutional computation. It adds the parameters of the convolution kernel to extract the features of the data. The parameters of the convolutional layer include three parts: size, step size, and padding.

**Figure 1:**
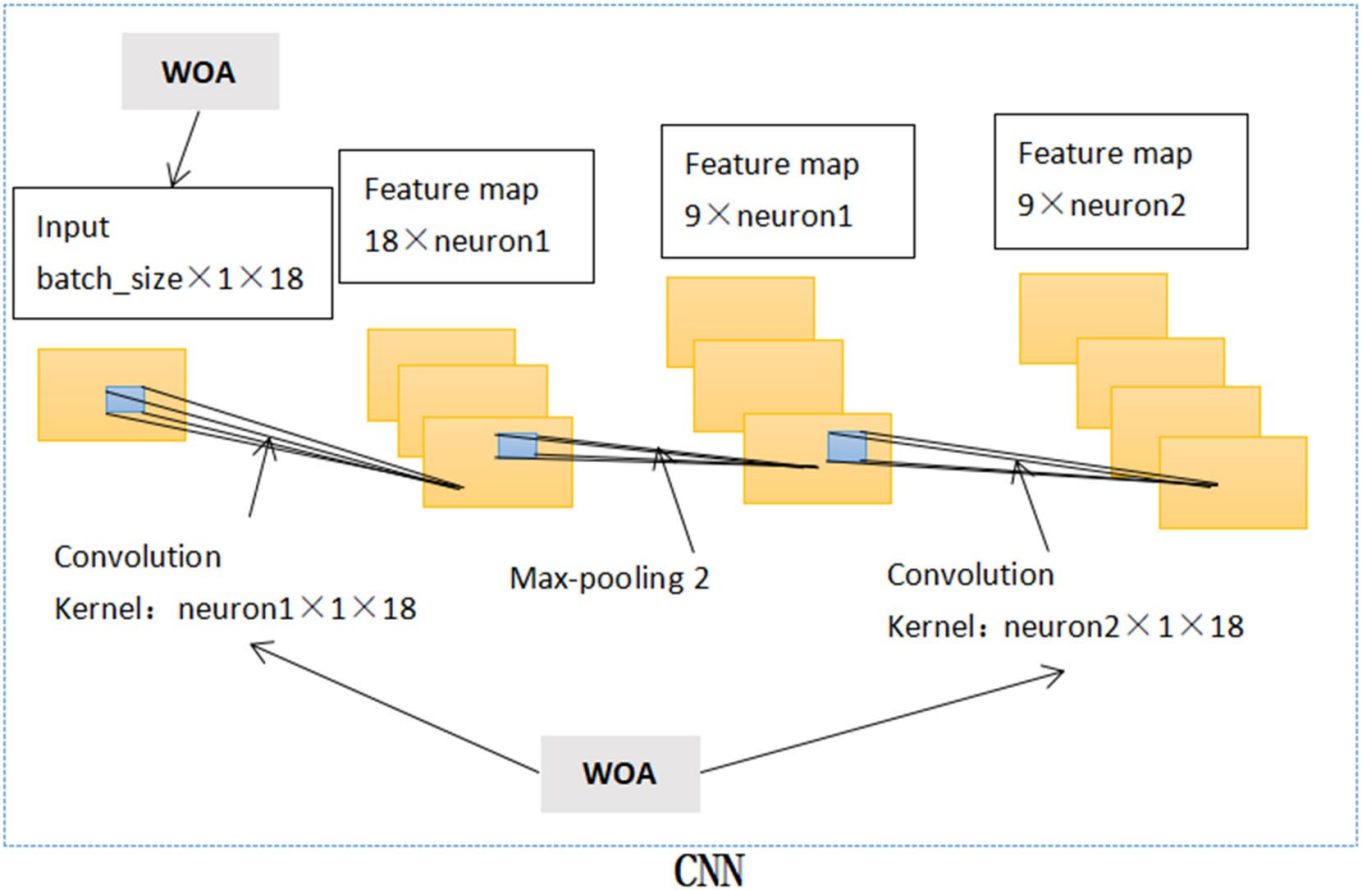
CNN internal structure of the WOCLSA.

**Figure 2:**
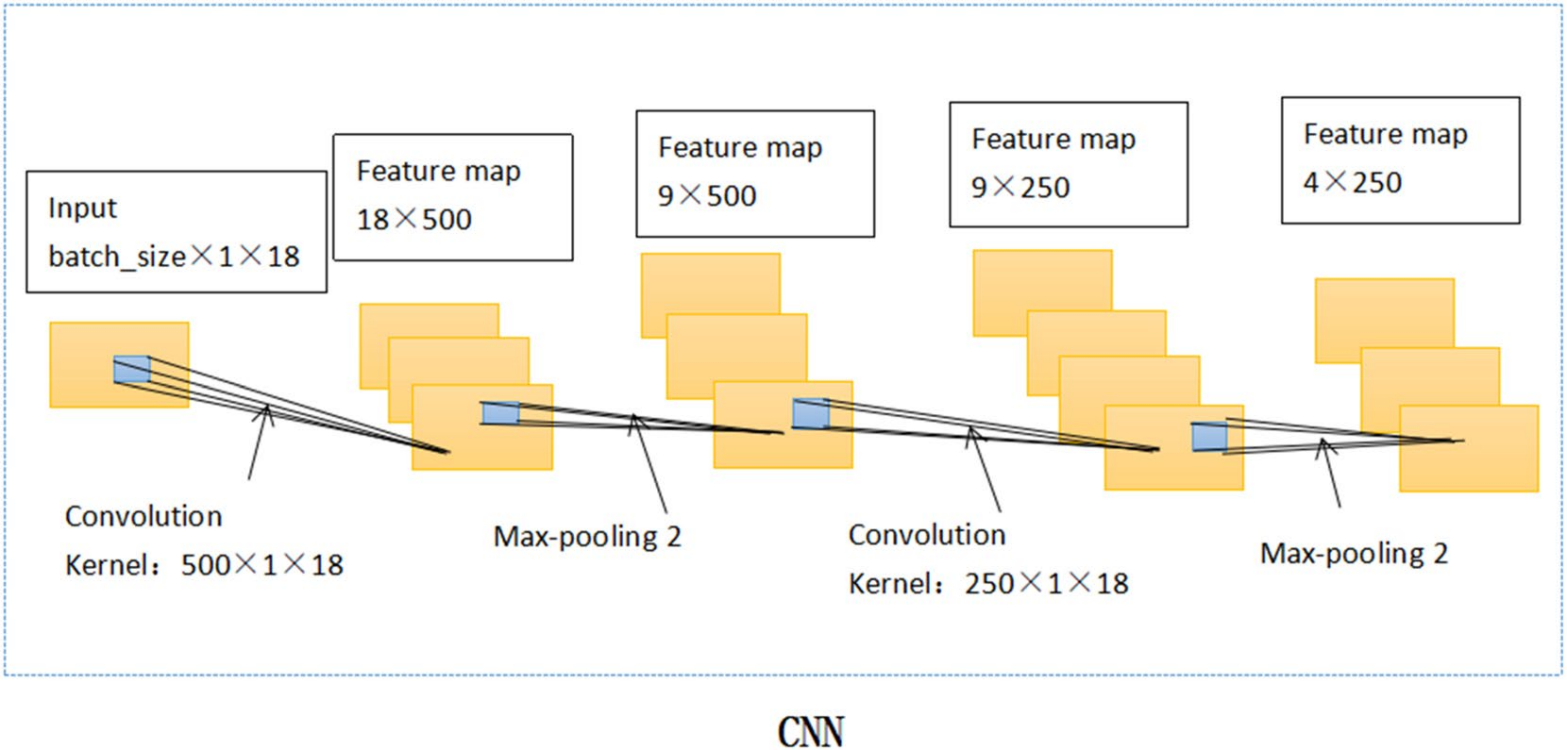
CNN internal structure of CNNLSTMA and CNNRNNA models.

**Figure 3:**
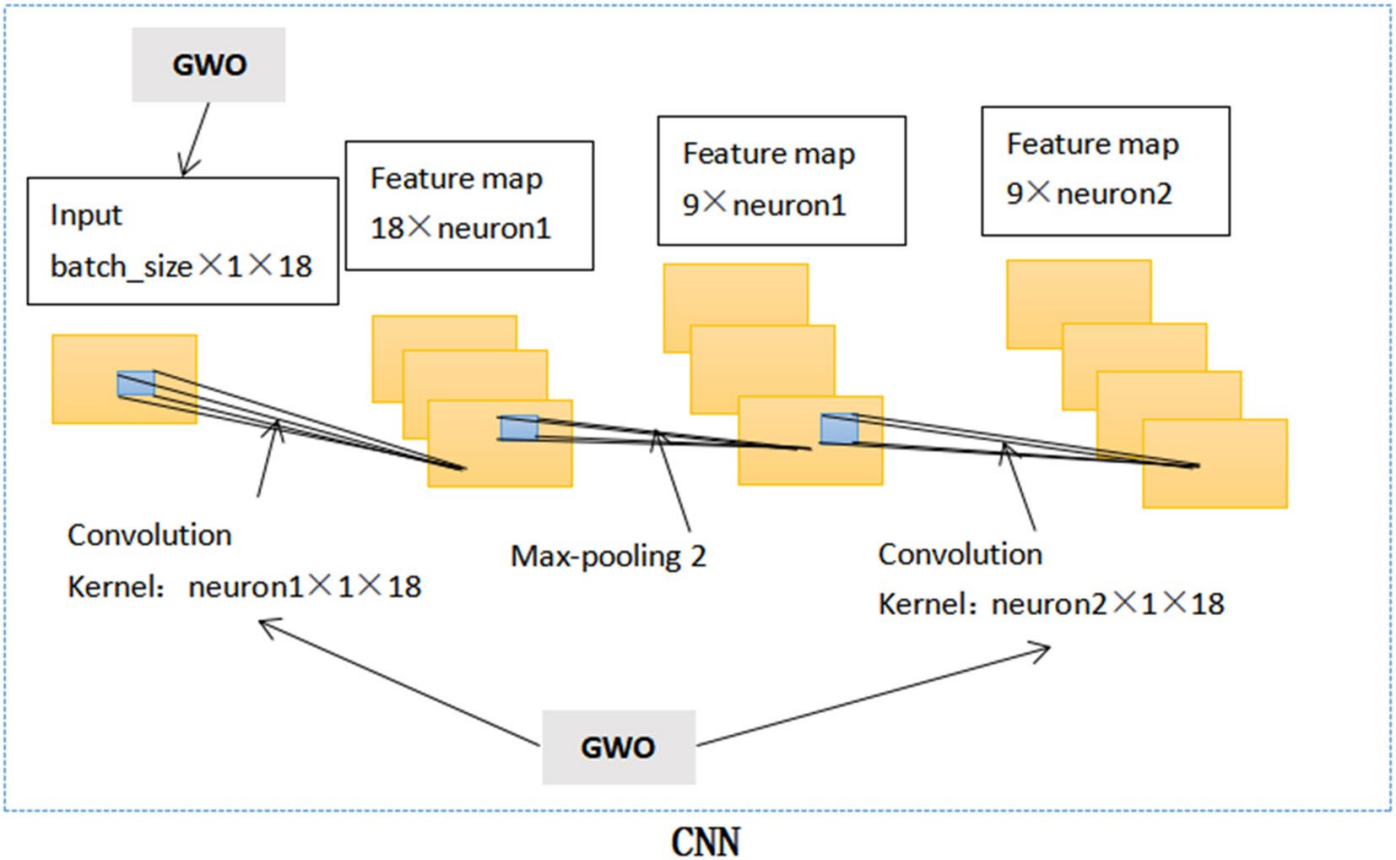
CNN internal structure of the GWOCLSA deep learning model.

Undoubtedly, these parameters above are crucial. They jointly determine the output characteristics of CNN. In this paper, the training data from 480 patients was processed by batch size and entered into the deep learning model in batches. The amount of batch size is determined by WOA. Figure 1 shows the CNN internal structure of the WOCLSA deep learning model. CNN does the first convolution to generate the feature map. The number of filters for the first convolution kernel is set to neuron 1. Feature maps are pooled through the pooling layer. Next, CNN does the second convolution. The number of filters for the second convolution kernel is set to neuron 2. Batch size, neuron 1 and neuron 2 use WOA to find the optimal parameter values. For the other two deep learning models, fixed parameters are adopted. The number of filters for the first convolution kernel is set to 500 and the number of filters for the second convolution kernel is set to 250. Batch size is set to the default parameter. Figure 2 shows the CNN internal structure of Convolutional Neural Networks (CNN), Long-Short Term Memory (LSTM) and Artificial Neural Network (ANN) (CNNLSTMA) and CNNRNNA deep learning models. Figure 3 shows the CNN internal structure of the GWOCLSA.

### LSTM

LSTM is a time recurrent neural network, which is an advanced version of Recurrent Neural Networks (RNN). LSTM has a more complex structure and has advantages in long sequence problems. It is divided into the following four departments:

First, LSTM uses the forgetting gate to determine the information carried by the cells on the upper layer. ${x}_{t}$ and ${h}_{t-1}$ are input and activated by the sigmoid function to obtain *f*. The output value ranges from 0 to 1.

1 $${f}_{{t}} = \sigma \left( {{W}_{{f}} \cdot \left[ {{h}_{{{t} - 1}} ,{x}_{{t}} } \right] + {b}_{{f}} } \right)$$

Then there is the update gate, which is divided into two parts. One is that this part can be seen as accepting new information, and the tanh activation function normalizes the content in [-1,1]. The other is that it can be seen as the content of new information retention.

2 $${i}_{{t}} = \sigma \left( {{W}_{{i}} \cdot \left[ {{h}_{{{t} - 1}} ,{x}_{{t}} } \right] + {b}_{{i}} } \right)$$

3 $${\tilde{C}}_{{t}} = {tanh}\left( {{W}_{{t}} \cdot \left[ {{h}_{{{t} - 1}} ,{x}_{{t}} } \right] + {b}_{{c}} } \right)$$

The following operation is to update ${c}_{t}$ and selectively forget the previous information to become a new ${c}_{t}$.

4 $$C_{t} = f_{t} *C_{t - 1} + i_{t} *\widetilde{{C_{t} }}$$

Finally, it is the output of LSTM. At this time, the neuron ${c}_{t}$ has been updated, using the ReLU activation function to output the content. The ${c}_{t}$ is scaled by tanh and multiplied by ${O}_{t}$. Next, put the cell state ${h}_{t}$.

5 $${ O}_{{t}} = \sigma \left( {{W}_{0} \left[ {{h}_{{{t} - 1}} ,{x}_{{t}} } \right] + {b}_{0} } \right)$$

6 $${h}_{{t}} = {O}_{{t}} {*tanh}\left( {{C}_{{t}} } \right)$$

### ANN

**Figure 4:**
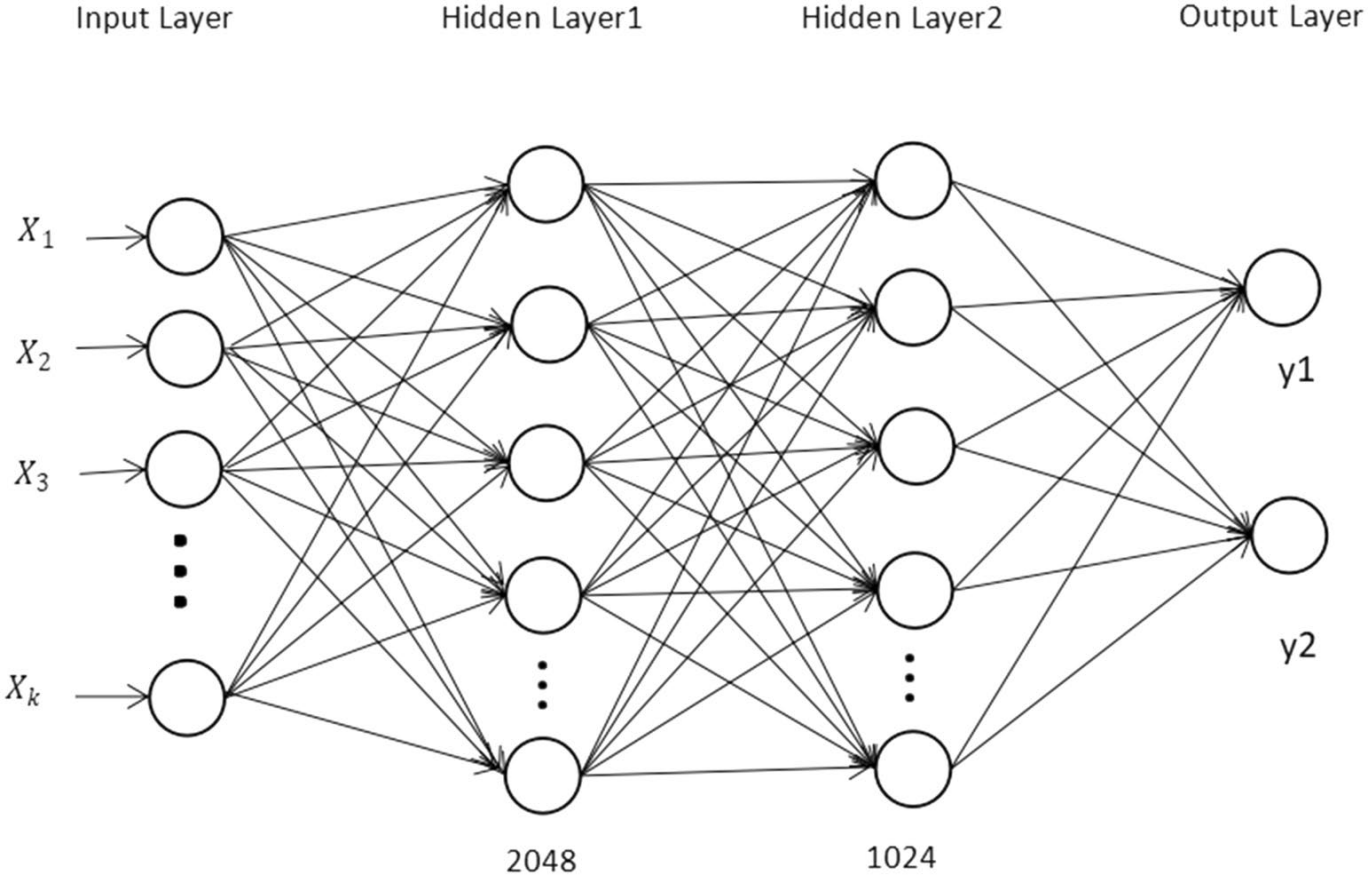
Basic structure of ANN.

ANN is a deep learning model that imitates the structure of the brain’s neural network. It contains a large number of neurons. When the input data enters the model, the weights of the neural network are updated by calculating the gradient of the error function through the back propagation algorithm, so that the output of the neural network is closer to the expected value. In this study, Fig. 4 is the structure of ANN. In the WOCLSA model, when the data is output from LSTM, the dimension of the output space is 512. The number of data entering the input layer is determined by the batch size. Figure 4 shows that there are k data sequences. We set the neuron parameters of hidden layer1 to 2048, and hidden layer2 to 1024 respectively.

### Generalization of WOA

Mirjalili and Lewis^32^ proposed the WOA. The WOA is a heuristic intelligent optimization algorithm that mimics the behavior of whale predation. If a whale finds prey, then other whales will surely swim to the whale that finds prey to compete for prey. In this paper, one solution represents a whale, and several solutions are represented by several whale individuals. In the process of using WOA to search for the solution of the problem, it can be seen that several whale individuals are constantly updating their individual positions until a satisfactory solution is found. The main behaviors include three parts: encircling prey, bubble-net attacking, and searching for predation. The following is the mathematical model of different stages of the WOA process.

#### Encircling prey

When the whale is encircling the prey, the whale position of the search agent will increase the number of iterations needed to update the position vector. These position vectors are related to the vector position of the best whale individual. We use a four-dimensional whale search dimension and 10 whale counts, setting the upper and lower limits of the search. In order to prevent the required parameters of neuron 1, neuron 2, dropout and batch size from overflowing, an adaptation function is set to correct these parameters.

7 $${Z} = \left| {{Q} \cdot {W^{*}} \left( {t} \right)} \right. - \left. {{W}\left( {t} \right)} \right|$$

8 $${W}\left( {{t} + 1} \right) = {W}^{*} \left( {t} \right) - {C} \cdot {Z}$$

where *t* represents the current iteration, *C* and *Q* represent the coefficient vectors, ${W}^{*}$ is the position vector of the optimal solution obtained in this way, and *W* represents the position vector of the whale. The values of *C* and *Q* are calculated according to the formula:

9 $$C = 2a \cdot r - a$$

10 $${Q} = 2{r}$$

As the number of iterations increases, ${\varvec{a}}$ decreases linearly from 2 to 0, and **r** is a random vector in [0,1].

#### Bubble-net attacking

Bubble net attack is the bubble predation behavior of whales. The attack of the bubble net is divided into contraction encirclement and spiral update position mechanisms. In order to realize the contraction surround behavior, it is almost the same as the mathematical model of the prey behavior mentioned above. The difference is that the value of *C* changes from [−a, a] to [−1,1]. In the stage of spiral update position mechanism, the search agent approaches the target in a spiral shape. For the next iteration, probability selection will be used. If the probability is less than 0.5, the shrink-wrap mechanism is adopted. The probability is greater than 0.5, and the spiral update position is used.

11 $${Z}^{\prime } = \left| {W^{*} \left( {t} \right) - \left. {{W}\left( {t} \right)} \right|} \right.$$

12 $${W}\left( {{t} + 1} \right) = { Z}^{\prime } \cdot { }e^{bk} \cdot { }\cos 2\pi k + { W}^{*} \left( {t} \right)$$

where W* is the position vector of the best search agent, *W(t)* is the position vector of the search agent in iteration *t*, *Z '* is the coefficient vector. b is a constant value. Random numbers are represented by *k*, and the range is [−1,1].

#### Search for prey

At this stage, if the predation of *C* does not belong to [−1,1], then the search agent whale randomly selects one from the current whale population instead of being close to the best whale agent. In order to avoid falling into the state of local optimal solution. The mathematical model is as follows:

13 $$Z = \left| {{Q}*{W}_{{{rand}}} - \left. {{W}\left( {t} \right)} \right|} \right.$$

14 $${W}\left( {{t} + 1} \right) = { W}_{{rand }} - {C} \cdot {Z}$$

$Wrand$ is the randomly selected location of the whale, $W(t)$ is the position of the search agent after t iterations. This paper has a total of three iterations. *C* and *Q* are position vectors, and *Q* is the coefficient vector^33^.

### Generalization of GWO

The Grey Wolf Optimizer (GWO) is also a swarm intelligence algorithm. The algorithm is an optimization search method developed to mimic the prey predation of gray wolves. The GWO process consists of three main steps: encircling, hunting, and attacking the prey^34^. The whale optimization algorithm is similar to the gray wolf optimization algorithm. Comparing with the whale optimization algorithm, we also set up 10 Gy wolves and had a total of three iterations. The range of prey searched by the gray wolf is also set to 4-dimensional.

### WOA algorithm optimizes deep learning model

Deep learning methods generally involve the following stages:

1. Data pre-processing.
2. CNNLSTM architecture update and reconstruction.
3. Performing model learning and data fitting.

The last stage is crucial for parameter selection. In this paper, we optimize some parameters using WOA such as dropout or batch size^10^.

In this paper, the number of training samples is small. In order to prevent the overfitting phenomenon which leads to distortion of prediction results, we apply the dropout function, which will enhance the generalization of the model. To explore the impact of the dropout function on the prediction accuracy and mitigate the risk of overfitting, this study employs the whale algorithm to optimize the parameters of the dropout function. This approach aims to identify an appropriate initial value that can lead to more consistent results. In addition, the size of batch size affects the reliability and efficiency of the model. It is not appropriate for the batch size to be too large or too small. Setting a reasonable batch size in the model can improve the training speed and make the gradient’s descending direction more accurate. Therefore, this model also optimizes the batch size parameter.

**Figure 5:**
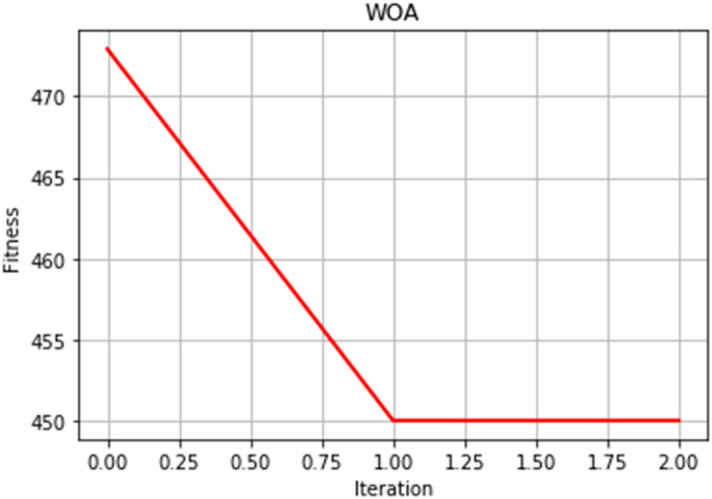
Optimal fitness curve diagram.

**Figure 6:**
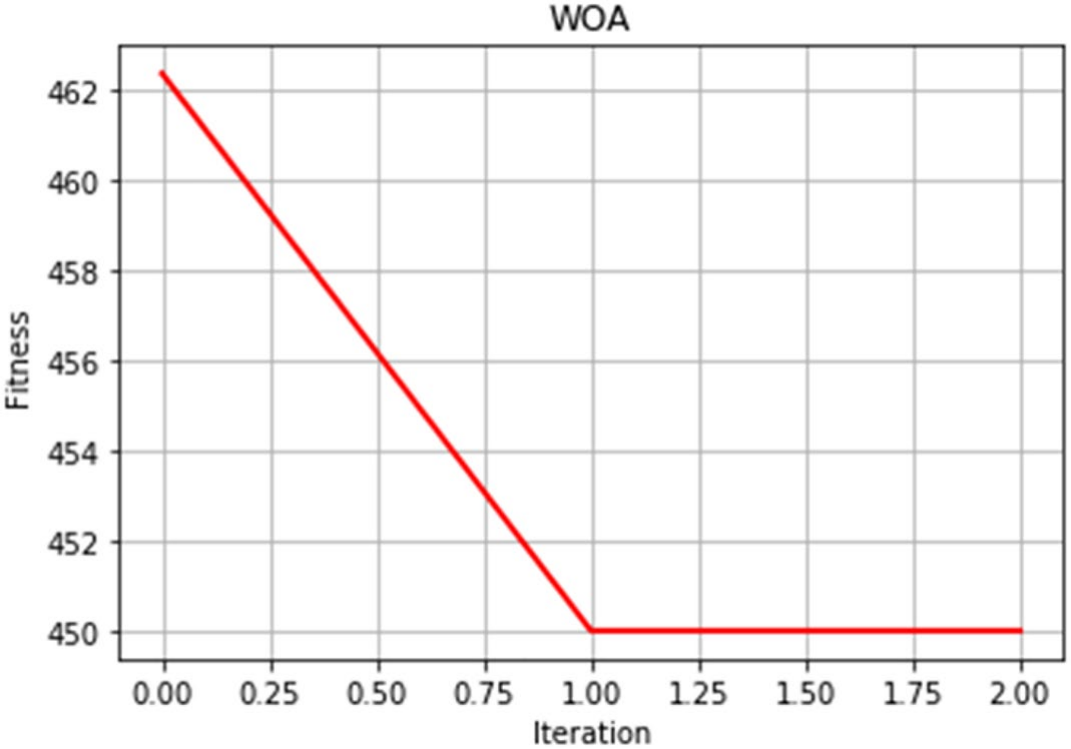
Optimal fitness curve diagram.

The convergence speed of the optimal fitness curve is a key indicator to evaluate the excellence of the WOA. The smoother and better the optimal fitness curve, the faster can converge to the optimal solution quickly. We adjust a four-dimensional whale search dimension. This experiment sets the whale search range, iteration times, and population number. After many comparative experiments, a target value close to the actual optimal value of the function is found. Figures 5 and 6 selected two experimental results.

## Innovating the framework of forecasting model

### Three ensemble models

**Figure 7:**
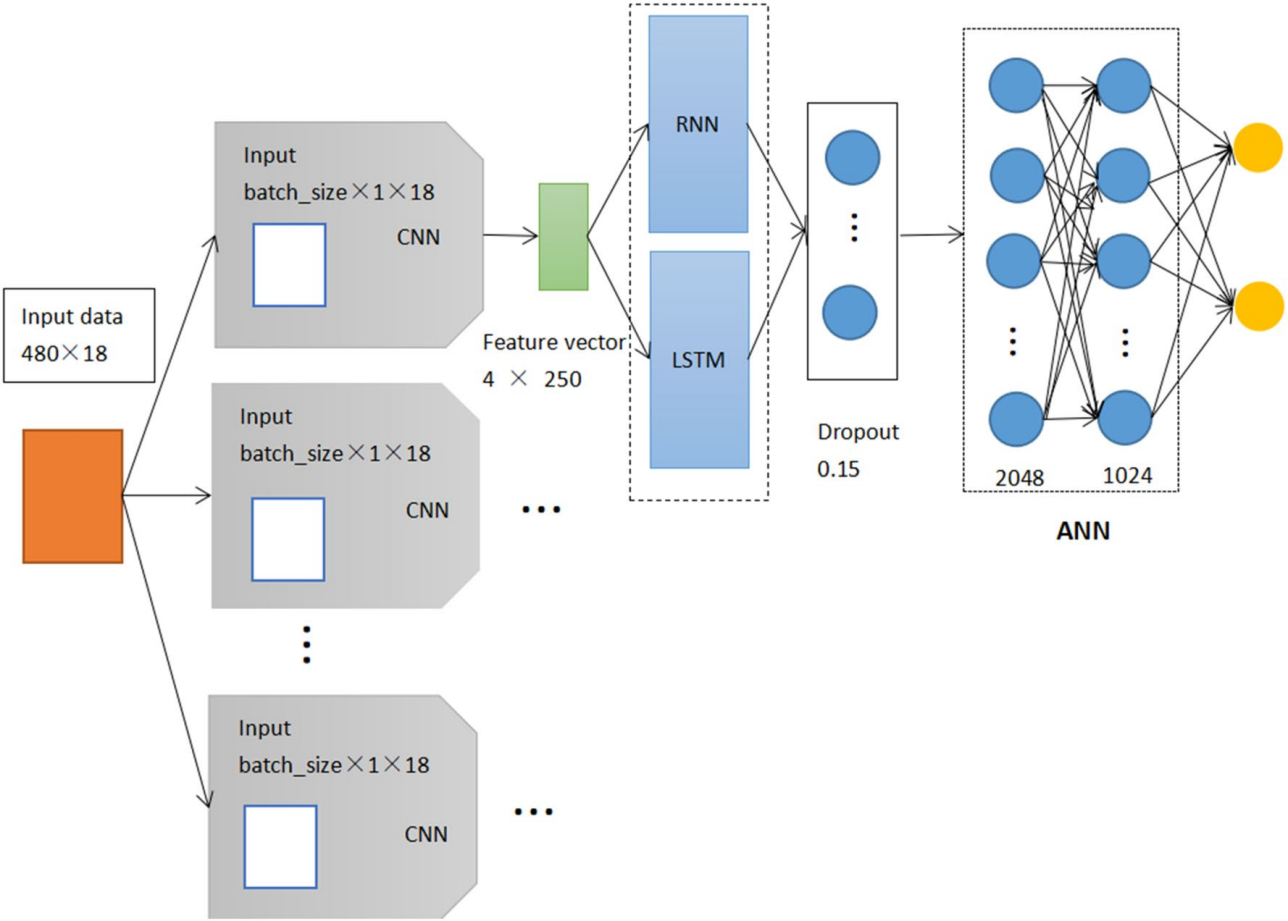
The basic structure of the CNNRNNA and CNNLSTMA.

In general, some models such as CNN and Recurrent Neural Networks (RNN) are used to predict medical diseases^35^. RNN is a kind of recursive neural network that uses sequence data as input. RNN solves the input problem through its internal memory, and all using the same functions in the process. The integrated model Artificial Neural Network (ANN), Convolutional Neural Networks (CNN) and Recurrent Neural Networks (RNN) is called CNNRNNA, and the data results are continued into the RNN model to run after the CNN completes its convolution^36,37^. Then, the LSTM dimension of the output space enters into neural network learning. Compared with classical RNN, LSTM can alleviate the gradient disappearance or explosion phenomenon of RNN. In addition, CNNLSTM has shown good prediction results due to this integrated model in Alakus and Turkoglu’s^31^ prediction, CNNLSTM combines the respective spatial and temporal advantages of ANN, CNN and LSTM, with the flexibility and efficiency of application in predicting public health diseases^38–42^. In this paper, the mixed model is obtained by adjusting the parameters of CNNLSTM model, which is used as a comparative prediction of the WOCLSA model, and the mixed model is called CNNLSTMA which includes CNN, LSTM and ANN. Therefore, the effects of CNNRNNA and CNNLSTMA mixed models are worth studying. Therefore, two hybrid models were used as comparative study experiments, Fig. 7 shows the basic structure of the CNNRNNA and CNNLSTMA.

**Figure 8:**
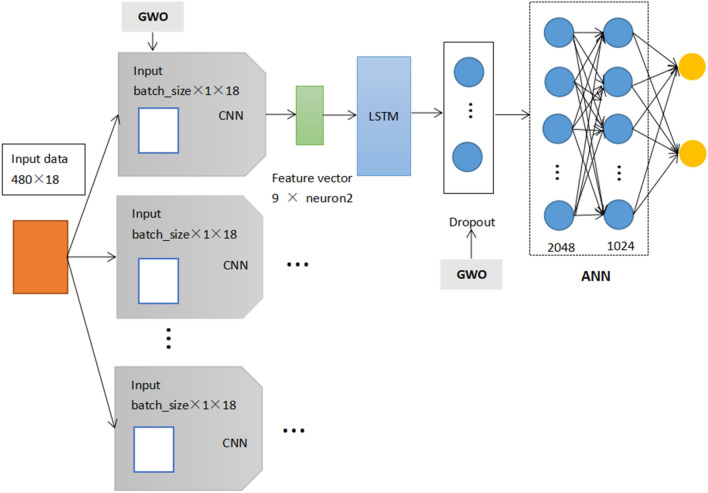
The basic structure of the GWOCLSA.

We use the Gray Wolf Optimization Algorithm to optimize the neuron number, dropout and batch size parameters of CNNLSTM and change the model structure of CNNLSTM. It is referred to as the GWOCLSA deep learning model. In order to better verify the prediction performance of the WOCLSA model, we added the GWOCLSA deep learning model as a comparison of heuristic optimization algorithms. Figure 8 shows the basic structure of GWOCLSA.

### WOCLSA

**Figure 9:**
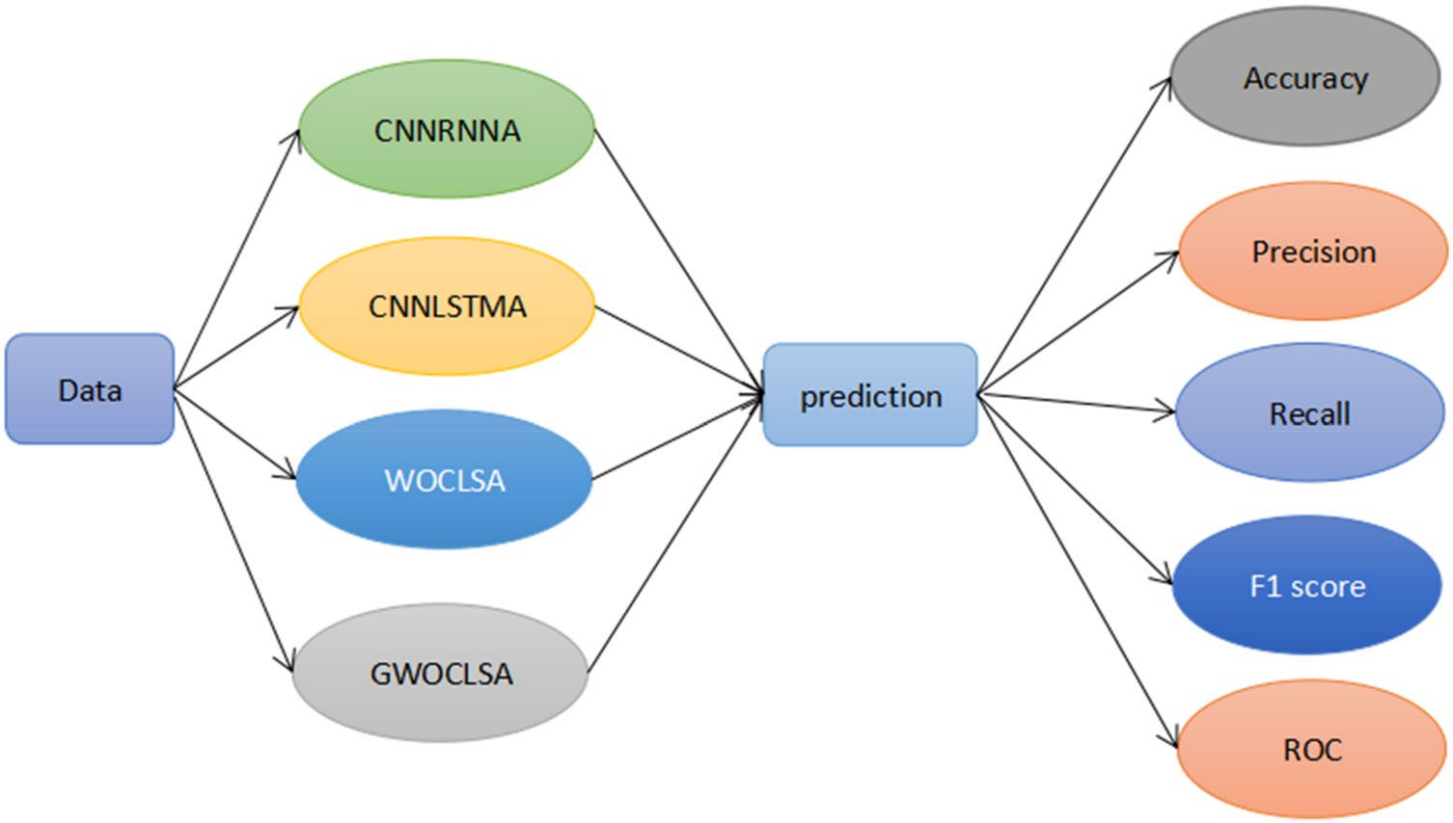
Flowchart of this study.

In this paper, the patient's data is input into four different deep learning models respectively, predicting the performance of these models and analyzing the results with five different evaluation metrics. Figure 9 images the flowchart of this paper.

**Figure 10:**
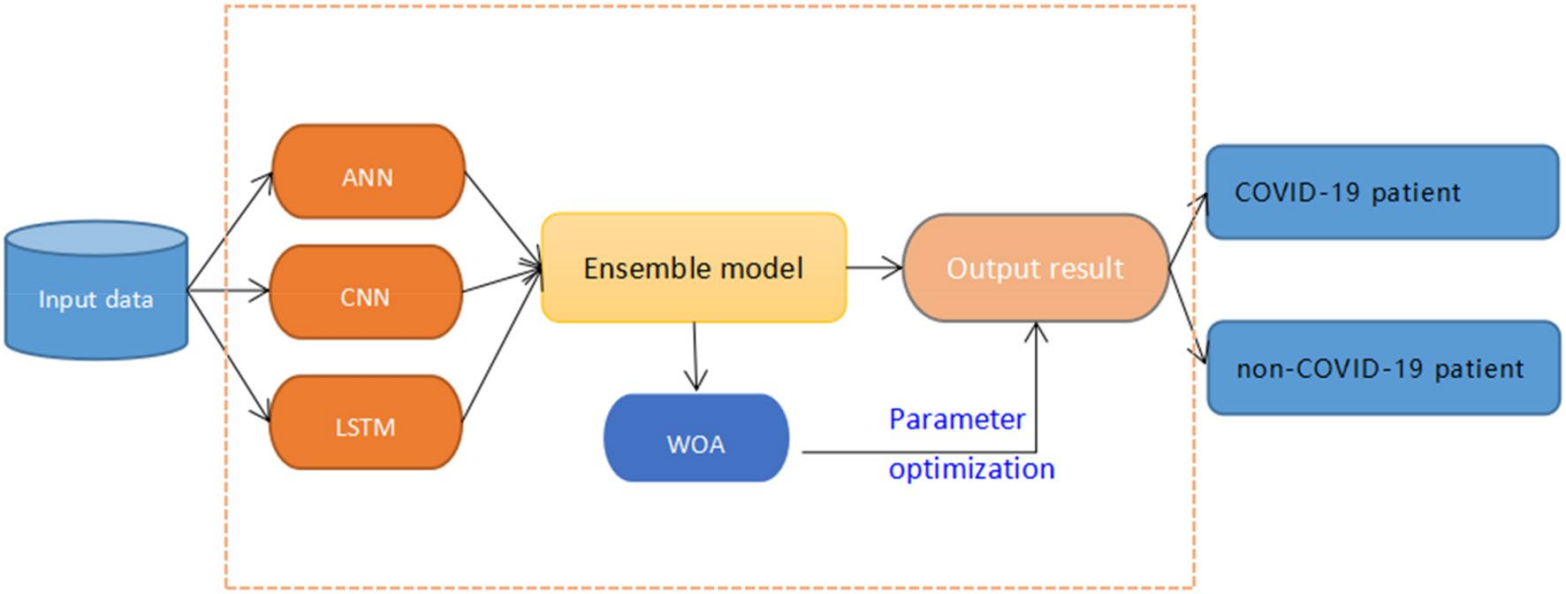
The framework of the WOCLSA prediction model.

In the WOCLSA deep learning model, the patient's data is fed into the model that ANN, CNN, and LSTM monolithic models are architecturally integrated. After the whale algorithm optimizes parameters of the integrated model, the output values are available in the classifier after the activation function to get the prediction results. Figure 10 shows the framework of the WOCLSA prediction model.

**Figure 11:**
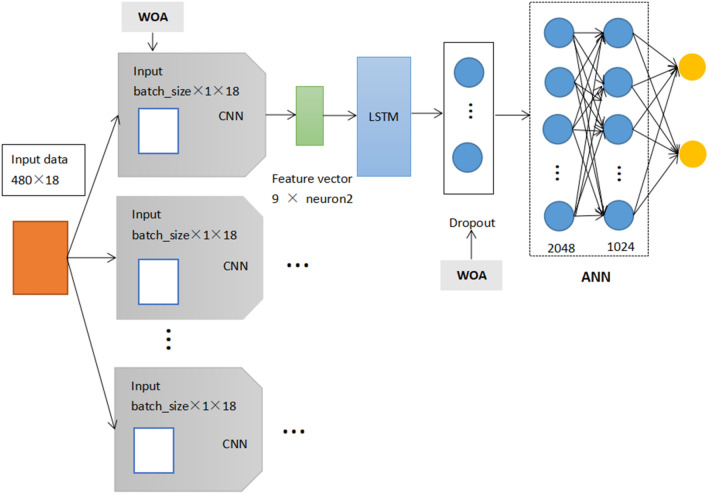
Detailed design diagram of WOCLSA.

Based on Alakus and Turkoglu’s^31^ study, the parameters in the original model were set for each model using the trial and error approach, which has certain errors and lacks universality. In this study, the CNNLSTM architecture was rebuilt and the parameters of dropout and the batch in the CNNLSTM model were optimized by WOA algorithm to find the global optimal solution parameters. When the complete data set is run through the model once and the results are returned once, this process completes an epoch. For all the integrated models, we set 100 epochs. It can make the model performance best and reduce over-fitting. Figure 11 shows the detailed design of WOCLSA.

## Data description and evaluation criteria

### Data description

The dataset includes the laboratory findings of the patients seen at the Hospital Israelita Albert Einstein at Sao Paulo Brazil^43^. In the above data set, there are 5644 various patients with 111 laboratory findings. There are 10% positive patients in the data, where 18 indicators in the findings were important for COVID-19 disease, as shown in Table 1 below, and after the balancing process, there are 18 laboratory findings from 600 patients left^31^. In the balanced dataset, we have 520 non-COVID-19 patients and 80 COVID-19 patients. Our experiment divides 600 patients into test and training subsets, where the training subset contains 480 patients and the test subset contains 120 patients. In cases of relatively small samples, k fold cross-validation approach and train-test split approach are used in health studies. Based on Comparison of deep learning approaches to predict COVID-19 infection the paper, for the hybrid model, tenfold cross validation prediction performance results are slightly lower than the train-test split method^31^. Therefore, we use 80–20 train-test split approach to validate the data. Table 1 below shows the indicators.

**Table 1:** Patient indicators.

Patient indicators	Hematocrit, hemoglobin, platelets, red blood cells, lymphocytes, leukocytes, basophils, eosinophils, monocytes, serum glucose, neutrophils, urea, C reactive protein, creatinine, potassium, sodium, alanine transaminase, aspartate transaminase

### Evaluation standards

We predict the performance of the model through five methods. They are accuracy, f1 score, accuracy, recall, and area under the ROC curve (AUC)^31,44^ respectively. In this section, we will summarize each evaluation standard as follows:

Accuracy (*A*) refers to the number of correctly classified samples as a percentage of the total number of samples. *N* represents the sample of all predicted COVID-19.

15 $$\begin{array}{*{20}c} {A = \left( {{tp} + {tn}} \right) / N } \\ \end{array}$$

16 $$\begin{array}{*{20}c} {N = tp + tn + fp + fn} \\ \end{array}$$

In the above statement, *tp* represents true positive, *tn* represents true negative,* fp* represents false positive, and *fn* represents false negative. Accuracy is a key research characteristic that measures the proximity of sample parameters to the overall characteristics^45^. By demonstrating the accuracy of a study through appropriate metrics, researchers can establish the reliability of their findings. Higher accuracy values signify greater reliability.

Precision can be defined as the ratio of correctly predicted positive observations to the total predicted positive observations. It is a performance metric in neural networks.

17 $$\begin{array}{*{20}c} {P = tp / \left( {{tp} + {fp}} \right)} \\ \end{array}$$

Recall refers to the ratio of predicted correct positive cases to actual positive cases.

18 $$\begin{array}{*{20}c} {R = tp / \left( {tp} + {fn} \right)} \\ \end{array}$$

In this paper, recall rate is the ratio of correctly identified COVID-19 patients to the total number of COVID-19 patients. The closer the recall score must be to 1 in order to be perfectly classified.

F1 score is the weighted average of precision and recall values. In other words, The higher the F1 score, the better the prediction effect.

19 $${F}1 = 2 / \left( {1{ }/{ P } + 1{ }/{ R}} \right) = { }2{ *P *R} / \left( {{P} + {R}} \right)$$

Finally, the AUC score has an important role in medical research. AUC is a measure of the classifier’s ability to distinguish between positive and negative classes. In order to predict the disease better, the closer the above evaluation criteria are to 1.

## Experimental results and analysis

### Experimental equipment

During all the experiments, we use the Spyder (3.2.3) development tool and employ the Python 3.6 programming language to execute in Tensorflow (1.2.1) exploitation environment. Our development code is based on the program provided by the author of Alakus and Turkoglu^31^.

### Results and analysis

In order to compare the time execution efficiency of the WOCLSA and GWOCLSA, we select the results of three experiments to study. The following conclusions can be drawn from Table 2, the execution time of WOCLSA is less than GWOCLSA every time. Therefore, WOCLSA is relatively efficient.

**Table 2:** The execution time of two deep learning model.

	Model	Total time
First time	WOCLSA	**94 min**
	GWOCLSA	108.9 min
Second time	WOCLSA	**98.2 min**
	GWOCLSA	121.6 min
Third time	WOCLSA	**99.8 min**
	GWOCLSA	109.6 min

Significant values are in bold.

We have performed many test experiments, the above are the results of three tests. From Table 3, we can see that after the parameter adjustment of the whale optimization algorithm, the area under the ROC curve has been significantly improved. At the same time, the prediction rate has been significantly enhanced, with the accuracy, F1 score, precision rate and recall value all exceeding 92%. The experimental value of WOCLSA exceeds that of other hybrid models. Although AUC of GWOCLSA is 93%, it is slightly higher than WOCLSA, the other evaluation indicators for GWOCLSA are below WOCLSA. From Table 4, the accuracy, F1 score, precision rate and recall value of the WOCLSA model is respectively 92.79%, 92.79%,92.79% and 92.79%. In the Table 5, these evaluation standards are still the highest compared to other models. The AUC values of the GWOCLSA and WOCLSA models are close based on the results of multiple tests.

**Table 3:** Evaluation standards of all ensemble models with train-test split approach in test 1.

	Accuracy	F1-score	Precision	Recall	AUC
CNNRNNA	0.8655	0.8655	0.8655	0.8655	0.74
CNNLSTMA	0.8657	0.8742	0.8742	0.8742	0.83
WOCLSA	**0.9282**	**0.9341**	**0.9341**	**0.9341**	**0.91**
GWOCLSA	0.9003	0.9160	0.9160	0.9160	0.93

Significant values are in bold.

**Table 4:** Evaluation standards of all ensemble models with train-test split approach in test 2.

	Accuracy	F1-score	Precision	Recall	AUC
CNNRNNA	0.8655	0.8655	0.8655	0.8655	0.74
CNNLSTMA	0.8789	0.8788	0.8788	0.8788	0.85
WOCLSA	**0.9279**	**0.9279**	**0.9279**	**0.9279**	**0.91**
GWOCLSA	0.9191	0.9211	0.9211	0.9211	0.90

**Table 5:** Evaluation standards of all ensemble models with train-test split approach in test 3.

	Accuracy	F1-score	Precision	Recall	AUC
CNNRNNA	0.8585	0.8535	0.8535	0.8535	0.79
CNNLSTMA	0.8890	0.8901	0.8901	0.8901	0.89
WOCLSA	**0.9166**	**0.9233**	**0.9233**	**0.9233**	**0.93**
GWOCLSA	0.9099	0.9066	0.9066	0.9066	0.94

Significant values are in bold.

**Figure 12:**
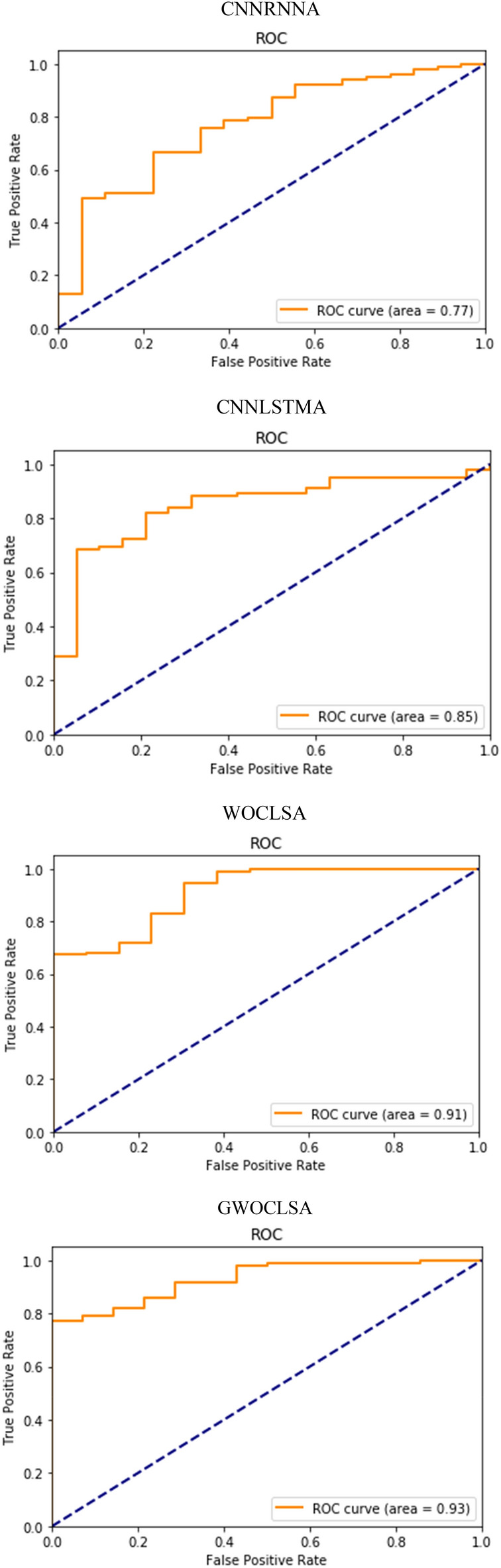
AUC values of all models.

**Figure 13:**
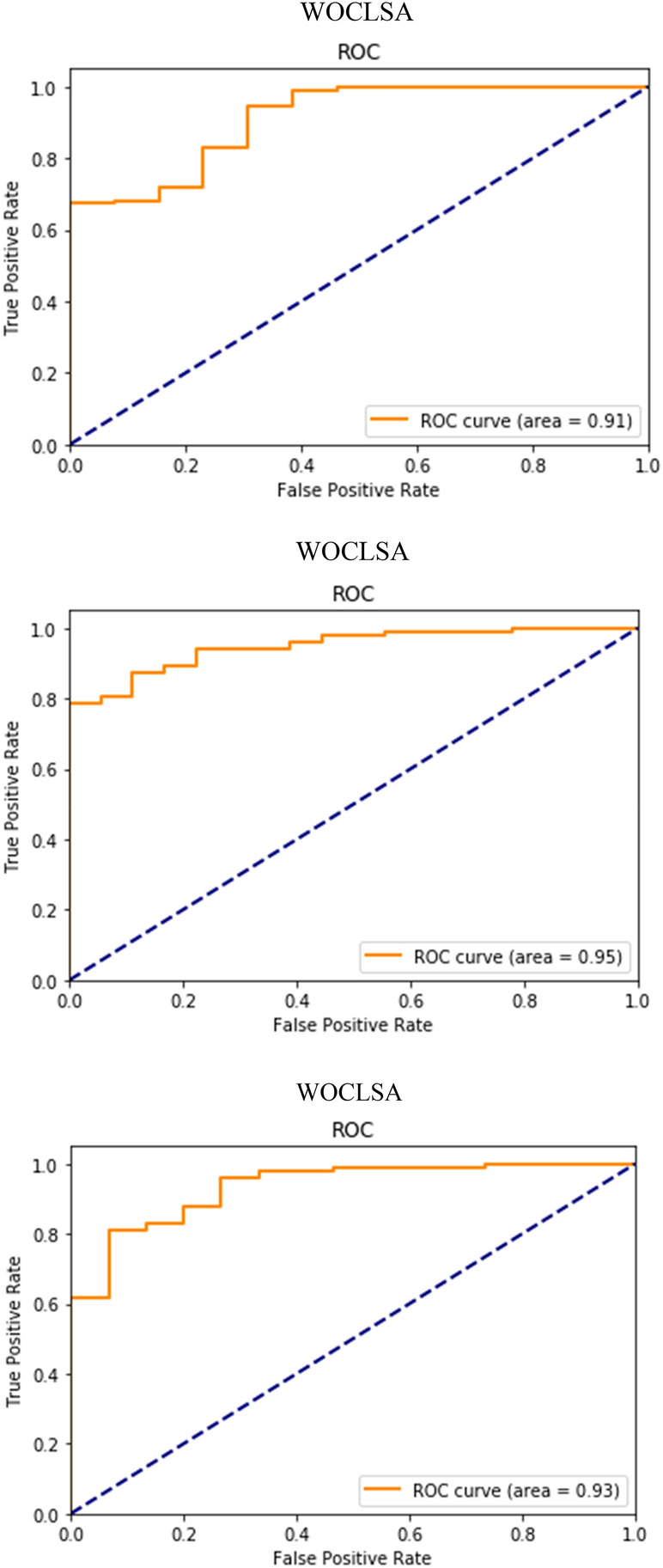
AUC values for multiple tests of the WOCLSA model.

From Fig. 12, an AUC score over 90% is considered outstanding. WOCLSA achieved good results, it can be seen that it reached 91%. The AUC of GWOCLSA is 93%, while the other two hybrid models by AUC scores were less than 90%. They do not play a good prediction effect. Figure 13 shows the AUC values for multiple tests of the WOCLSA model. The AUC has a high score of 91%, 95%, and 93%. The AUC of the WOCLSA and GWOCSA models are higher than that of CNNLSTM model researched by Alakus and Turkoglu^31^, which is 90%. Therefore, this is an innovative breakthrough in predictive performance. There will be some other factors in the process of prediction, such as the configuration of the computer and the nature of the errors in the calculated scores during the optimization of the heuristic algorithm. Although each prediction result is subject to small changes by these external factors, the overall high performance of the prediction is constant. The value of AUC plays a key role in medical disease classification^45^, a higher AUC score makes a more accurate prediction for real patients.

## Discussion

It is vital to use artificial intelligence systems to predict the clinical course of public health diseases. Various intelligent prediction models have been applied in numerous fields, providing medical professionals with valuable insights into clinical diagnostics. The country 's prediction of public health disease events has always attracted much attention. At present, the simulation research on the clinical prediction of COVID-19 is very limited.

For example, Zongxi et al.^46^ applied Gray Wolf optimization and multi-machine learning to predict COVID-19. The optimal weights of the ANFIS, LSSVM and LSTM models are searched by the Grey Wolf optimization algorithm, and the prediction model is established. Although machine learning is used in most studies of prediction in medicine, the predictive performance of deep learning still deserves to be explored and applied. The prediction accuracy of deep learning model has been improved, but it has been used to predict the epidemic trend in COVID-19, rather than clinical diagnosis. There are some models such as IPSO–DNN and MPSO–CNN that optimize parameters with intelligent algorithms^47,48^. Sun et al.^49^ used a Genetic algorithm to optimize ANN. However, these models are relatively backward, and the prediction accuracy is low. We also have different evaluation criteria for predicting diseases from these models. In addition, Qian et al.^50^ and Pasaribu et al.^51^ respectively applied the Infectious disease dynamics model (SEIR) and the big data Model (GSTAR).

From Tables 3, 4, and 5, it can be seen that the prediction performance of the deep learning model optimized by whale algorithm has high reliability. As an optimization comparison of heuristic algorithm, the prediction performance of grey wolf algorithm is lower than that of whale algorithm.

At present, the lack of literature research includes the following three factors: there are few models for medical diagnosis based on patient laboratory indicators; most predict outbreak trends of COVID-19, rarely predict patient-specific disease; the prediction performance of existing deep learning models is low, and stable and efficient models are urgently needed.

Based on the above analysis, we propose an innovative ensemble model based on deep learning. The main contributions are as follows:

- Optimization of the internal parameters of the CNNLSTM using WOA, and reconstructing its architecture. As a heuristic algorithm, WOA is well combined with deep learning model. It has a better optimization effect than other heuristic algorithms.
- The ensemble model (WOCLSA) is proposed to improve the prediction performance. The evaluation criterion of the model are superior to those of other deep learning models. The AUC is 91%, which is higher than previous research results.
- The innovative ensemble model can be used to predict various public health diseases and provide help for medical systems and laboratory research. Meanwhile, WOCLSA model prediction method can be used for in other fields to study and explore.

## Conclusion

In the field of medical research, the accurate prediction of patients' diseases is a crucial component that enables effective clinical judgement and facilitates the improvement of treatment efficiency. A precise prediction can also help to alleviate the burden on hospitals and reduce the unnecessary waste of medical resources, which are often limited due to regional disparities in development. By prioritizing scarce medical resources, clinicians can ensure that essential epidemic prevention materials and emergency management strategies are deployed efficiently, thereby minimizing wastage and maximizing the benefits of available resources.

At present, most diseases are diagnosed by image recognition, and few studies are made by using patients' laboratory results. Research on COVID-19, most of them study the outbreak trend, and there is almost no research focused on personal laboratory indicators for medical diagnosis or laboratory research. Based on the research conclusion of Alakus and Turkoglu^31^, it is concluded that the prediction effect of mixed model is higher than that of single model, so we use different ensemble models for comparative forecasting. We compare the innovative ensemble model WOCLSA with the other three mixed models, and the performance of predicting COVID-19 infection is improved. It is a deep learning model lacking at present.

We employ a set of 600 data points for prediction. The results show the WOCLSA deep learning model outperforms other models in terms of prediction performance, with all evaluation criteria exceeding 90%. In terms of time efficiency, the program execution time of WOCLSA is less than that of GWOCLSA. Therefore, the WOCLSA model is able to enhance prediction performance and reduce the burden of doctors. In addition, the innovative ensemble model can reduce the error of manual diagnosis, provide a new public health disease model for prediction and play an important role in biomedical research.

For future research, the following directions can be pursued:

- Further studies could increase the sample of hospital patients and not be limited to the 600 laboratory study results. In this paper, the sample data used for prediction is relatively small. For the WOCLSA, the larger the dataset, the better it is to validate its predictive performance.
- Future studies should focus on optimizing index selection. This paper only considered 18 indicators, but medical diagnosis can benefit from additional or fewer indicators in the future.

## Data Availability

The balanced data set of laboratory findings is derived from the study of Alakus and Turkoglu. The balanced dataset via https://github.com/burakalakuss/COVID-19-Clinical.
